# The Electric Conductivity of Bi_7_Fe_3_Ti_3_O_21_ Doped with Gadolinium

**DOI:** 10.3390/mi15070860

**Published:** 2024-06-30

**Authors:** Jolanta Makowska, Diana Szalbot, Małgorzata Adamczyk-Habrajska, Beata Wodecka-Duś, Maciej Chrunik

**Affiliations:** 1 Institute of Materials Engineering, Faculty of Science and Technology, University of Silesia, 75 Pułku Piechoty 1A, 41-500 Chorzow, Poland; jolanta.makowska@us.edu.pl (J.M.); diana.szalbot@gmail.com (D.S.); beata.wodecka-dus@us.edu.pl (B.W.-D.); 2 Institute of Applied Physics, Military University of Technology, 2 Gen. Sylwestra Kaliskiego St., 00-908 Warsaw, Poland; maciej.chrunik@wat.edu.pl

**Keywords:** Bi_7-*x*_Gd*_x_*Fe_3_Ti_3_O_21_, Aurivillius phase, impedance spectroscopy

## Abstract

Bi_7-*x*_Gd*_x_*Fe_3_Ti_3_O_21_ (*x* = (0, 0.2, 0.4, 0.6)) bismuth-layered perovskite structure compounds have been successfully prepared by a solid-state reaction. The results of X-ray studies indicate that a single-phase ceramic was obtained, characterized by an orthorhombic crystal structure for all compounds within the *Fm*2*m* space group. Microstructural analysis revealed that introducing gadolinium to the material altered the grain morphology, resulting in a more rounded grain shape and a somewhat disordered arrangement. Moreover, with higher gadolinium concentrations, there is a noticeable increase in the presence of the number of large plates. Impedance spectroscopy has been used to characterize the electrical properties of Bi_7-*x*_Gd*_x_*Fe_3_Ti_3_O_21_ compounds.

## 1. Introduction

The modern world and its rapid technological progress has forced researchers to develop innovative materials to keep pace with civilization’s progress. One example is multiferroics, which simultaneously exhibit ferromagnetism, ferroelectricity, and ferroelasticity under the same phase conditions [1,2,3,4]. These materials have attracted the interest of researchers due to their interesting physical phenomena and significant application potential [5,6,7,8,9,10]. With order parameters such as electric polarization and magnetic ordering coexisting and interacting, they pave the way for innovative functionalities that could revolutionize electronic and spintronic devices [11,12,13]. A significant class of multiferroic materials is defined by the Aurivillius phase structure, which can be described by the general formula (M_2_O_2_)^2+^(A_*n*−1_B_*n*_O_3*m*+1_)^2−^ [14,15,16,17]. The structure of Aurivillius compounds consists of regularly arranged layers (M_2_O_2_)^2+^ and perovskite blocks (A_*n*−1_B_*n*_O_3*m*+1_)^2−^ [18], where n is the number of layers of these blocks. M sites are almost always occupied by bismuth ions [15], while the sublattice A is occupied by cations such as Bi^3+^, Sr^2+^, Ba^2+^, and Ca^2+^ [19], and the B sublattice by the ions Nb^5+^, Ta^5+^, W^6+^, and Mo^6+^ [20,21,22,23,24]. The wide array of possible ion substitutions within both the perovskite blocks and the bismuth–oxygen layers offers extensive possibilities for altering the physical properties of these compounds, enabling virtually limitless modifications of their characteristics. One of the most interesting materials within this group is Bi_7_Fe_3_Ti_3_O_21_ ceramics [25]. The composition of these six-layer compounds with an Aurivillius-type structure, as in the case of other members of the family, is extremely complicated [26,27]. This Aurivillius-type compound exhibits typical ferroelectric and ferroelastic properties and includes magnetic properties due to the presence of iron ions. The literature presents many interesting publications on multiferroism in Aurivillius structures; however, the Bi_7_Fe_3_Ti_3_O_21_ compound is poorly researched. The study of Bi_7_Fe_3_Ti_3_O_21_ and similar materials is crucial for advancing our understanding of the complex interactions between their structural and electronic properties, which is essential for the development of future multifunctional materials and their potential applications. The main application area focuses on utilizing multiferroic ceramics in energy-harvesting systems integrated with renewable energy sources, especially those connected to solar power. In multiferroic materials, electric and magnetic fields coexist. Moreover, these materials are characterized by a small energy gap. These features make them very attractive for photocatalytic applications [1]. The internal electric field, associated with the presence of polarization, may effectively help separate photo-generated electrons and holes. However, in the photocatalysis process, not only is the separation process necessary, but catalyst recycling is equally essential. The internal magnetic field originating from ferromagnetic properties could help make recyclable photocatalysts [1,2,3], and the small energy gap guarantees a better overlap with the solar spectrum [4].

Our previous paper concerned the dielectric and magnetoelectric properties of Bi_7-*x*_Gd*_x_*Fe_3_Ti_3_O_21_ ceramics, whereas the current article presents a comprehensive study focusing on the structure, microstructure, and impedance spectroscopy of these materials. In the global literature, there are no reports on the impact of gadolinium doping on the properties of Bi_7_Fe_3_Ti_3_O_21_ ceramics. Ceramic materials could be visualized as grain–grain boundary–electrode systems. Each microstructure component participates in the overall electrical properties of the investigated ceramic materials. In order to define the contributions, impedance spectroscopy is widely used. Analysis of the impedance spectroscopy results provides important information about the electrical properties of the investigated materials, including helping to determine the mechanisms of electrical conduction in the grains and the grain boundaries, which is essential from the perspective of fundamental research and to determine the material’s potential applications.

## 2. Materials and Methods

Bi_7-*x*_Gd*_x_*Fe_3_Ti_3_O_21_ (*x* = (0, 0.2, 0.4, 0.6)) ceramics were synthesized using the solid-state reaction and the conventional mixed oxides method. Stoichiometric quantities of bismuth oxide (Bi_2_O_3_, Aldrich 99.9%, St. Louis, MO, USA), titanium dioxide (TiO_2_, Poch 99.9%, Gliwice, Poland), and iron oxide (Fe_2_O_3_, Sigma-Aldrich 99%, St. Louis, USA) were initially combined and mixed in a mortar for 45 min. The mixture was then ground in a planetary mill at 250 r/min for t = 24 h with ethanol and yttria-stabilized zirconia balls, followed by drying and mixing in the mortar for t = 30 min. The following equation was used (1):

(1) $$\left(3.5-0.5x\right){Bi}_{2}O_{3}+1.5{Fe}_{2}O_{3}+3TiO_{2}+\frac{x}{2}{Gd}_{2}O_{3}\to {Bi}_{7-x}{Gd}_{x}{Fe}_{3}{Ti}_{3}O_{21}$$

The synthesis reaction occurred at T = 1123 K for t = 4 h in a muffle furnace. Post-synthesis, the powder was re-ground, pressed into pellets (d = 10 mm, h = 1 mm) at p = 200 MPa, and sintered in closed corundum crucibles at T = 1263 K for t = 4 h. The relative density was obtained using the Archimedes method. For SEM/EDS tests, the surfaces of the samples were coated with gold to provide electrical conductivity. For impedance spectroscopy measurements, the opposing pellet faces were covered with platinum electrodes (coated using the firing method).

The crystal structures of all ceramic materials were determined using a BRUKER D8 Discover (Karlsruhe, Germany) X-ray diffractometer, emitting standard characteristic radiation from a CuKα copper anode (λKα1 = 1.54056 Å, λKα2 = 1.54439 Å, Siemens KFL CU2K, with lamp operating parameters U = 40 kV and I = 40 mA). Crystallographic calculations of the ceramic samples were performed using the Rietveld method via the FullPROF version 3.0 software [28], which is utilized for the analysis of X-ray diffraction profiles, enabling the precise calculation of unit cell parameters for all examined materials based on diffraction profile fitting (ICSD 155931). 

Microstructural analysis was performed with a JEOL JSM-7100F TTL LV (Tokyo, Japan) scanning electron microscope equipped with an energy dispersion spectrometer (EDS), using X-ray microanalysis for both qualitative and quantitative chemical composition assessments.

Impedance spectroscopy measurements were conducted using an Agilent E4980A impedance analyzer with a frequency range from 20 Hz to 2 MHz and at temperatures ranging from T = 475 to 800 K. All impedance data underwent a consistency test based on the Kramers–Kronig (K-K) equations. A detailed description of the data consistency testing method used can be found in [29]. The Kramers–Kronig test was performed using the K-K test software V1.01 [30,31], and a detailed analysis of the experimental data was conducted using ZView software (version 3.5, Scribner Associates, Inc., Southern Pines, NC, USA). 

## 3. Results and Discussion

### 3.1. X-ray Diffraction

**Figure 1:**
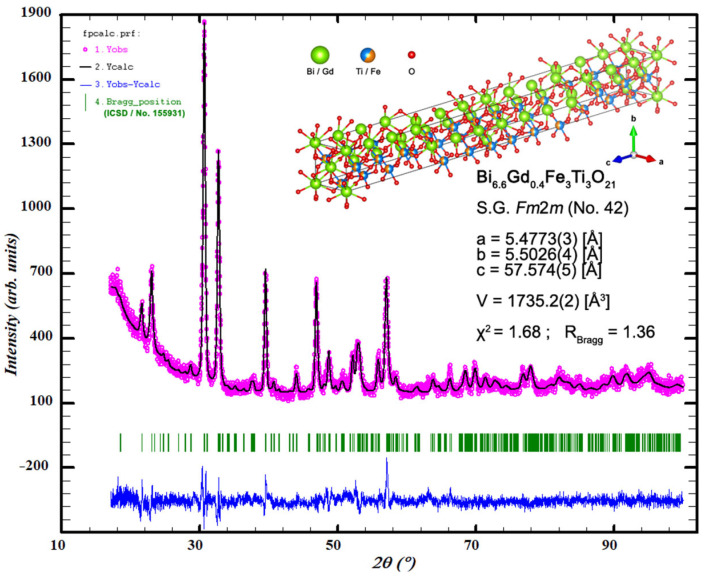
An exemplary pattern obtained from the Rietveld refinement of Bi_6.6_Gd_0.4_Fe_3_Ti_3_O_21_ powders (generated using WinPlotr).

In order to determine the crystal structure of the obtained Bi_7-x_Gd_x_Fe_3_Ti_3_O_21_ ceramic materials for x = (0, 0.2, 0.4, 0.6), X-ray analysis was carried out. Analyzing the obtained test results, it was found that all tested ceramic materials show phase homogeneity. Except for the target Aurivillius phase, neither residues of substrates nor intermediate phases from the oxide equilibrium systems (bismuth, iron, titanium, gadolinium) or other inclusions were recorded. Figure 1 shows an exemplary pattern obtained from the Rietveld refinement of Bi_6.6_Gd_0.4_Fe_3_Ti_3_O_21_ powders (generated using WinPlotr ver. April 2023 (as a part of FullPROF Suite 5.20, ver. December 2023)).

In the next stage of analysis of the X-ray study results, using the Rietveld method [32], the parameters of the unit cell (a, b, and c) and its volume (V) were determined based on the obtained X-ray spectra. Bi_7-x_Gd_x_Fe_3_Ti_3_O_21_ ceramics are characterized by an orthorhombic structure within the space group *Fm*2*m* and unit cell parameters for x = 0.2 - a_0_ = 5.4725(3) [Å], b_0_ = 5.4858(4) [Å], and c_0_ = 57.531(4) [Å]; for x = 0.4 - a_0_ = 5.4773(3) [Å], b_0_ = 5.5026(4) [Å], and c_0_ = 57.574(5) [Å]; and for x = 0.6 - a_0_ = 5.4695(3) [Å], b_0_ = 5.4978(5) [Å], and c_0_ = 57.591(8) [Å]. The volume of the unit cell is, respectively, - V = 1727.2(2) [Å^3^] for x = 0.2, V = 1735.2(2) [Å^3^] for x = 0.4, and V = 1731.8(3) [Å^3^] for x = 0.6 [33]. Analyzing the unit cell parameters, we see an increasing trend in volume changes correlating with a rise in the dopant levels. As the gadolinium content increases from x = 0.2 to x = 0.6 in the Bi_7-x_Gd_x_Fe_3_Ti_3_O_21_ structure, a slight expansion of the unit cell volume is noted at x = 0.4. This expansion is accompanied by an elongation of the lattice constant (b), which occurs alongside a less proportionate reduction in the lattice constant (a). In contrast, no significant or directional constant (c) changes are detected. The Rietveld refinement factors are summarized in Table 1.

### 3.2. Scanning Electron Microscopy and X-ray Microanalysis (EDS)

**Figure 2:**
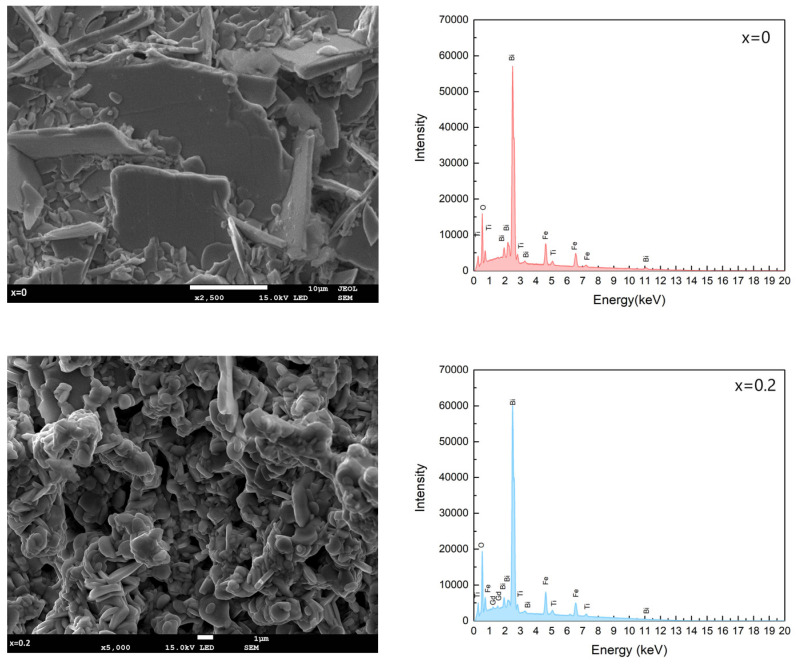

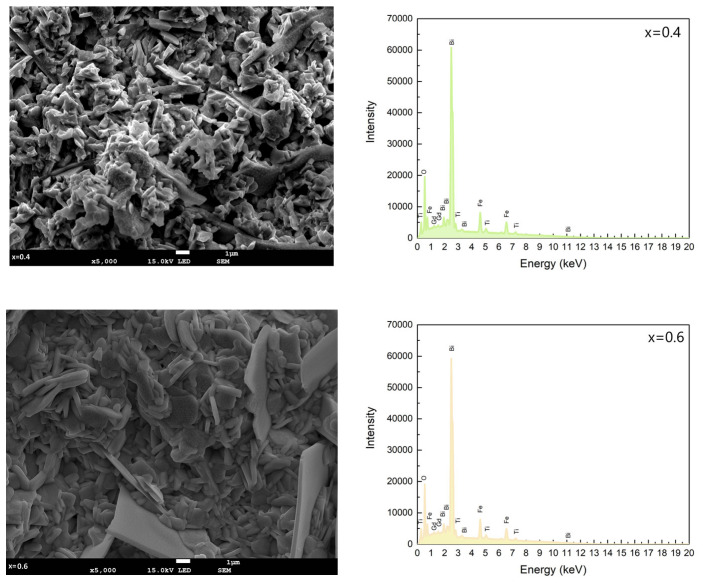
Microstructure of fractures of ceramic materials and EDS spectra of Bi_7-x_Gd_x_Fe_3_Ti_3_O_21_ ceramics for x = (0, 0.2, 0.4, 0.6).

Microstructural studies and chemical composition analyses were carried out on the fractures of the samples. In Figure 2, the microstructure of the fractures of the ceramic materials and the EDS spectra of Bi_7-x_Gd_x_Fe_3_Ti_3_O_21_ ceramics are presented for x = (0, 0.2, 0.4, 0.6).

In their earlier research, the authors noted that the grains in the ceramics that were not doped with Bi_7_Fe_3_Ti_3_O_21_ displayed a layered structure, aligning with the typical morphology observed in compounds that include Aurivillius phases [34,35]. Due to the large grain size of Bi_7_Fe_3_Ti_3_O_21_ ceramics, a magnification of 2500 was used. Increasing the amount of gadolinium significantly alters the fracture microstructure appearance in the studied ceramic materials. The plates are noticeably more rounded in the sample with a gadolinium content of x = 0.2, and the grain arrangement becomes quite chaotic. With a gadolinium admixture of x = 0.4, large plates start to emerge within the material, yet they are relatively few compared to the rest of the grains. Introducing a greater quantity of the modifier increases the proportion of larger plates. However, they do not become predominant. The disordered arrangement of grains is associated with the changes observed in the density of the examined materials. The density of the undoped ceramic constitutes 85% of the theoretical density, while with increasing gadolinium dopant content, this proportion amounts to 83%, 88%, and 94% of theoretical density (which was presented in our previous paper) [33]. EDS analysis confirmed the qualitative and quantitative chemical composition of the produced Bi_7-x_Gd_x_Fe_3_Ti_3_O_21_ ceramics and excluded the involvement of foreign elements or possible impurities. It can be concluded that Bi_7-x_Gd_x_Fe_3_Ti_3_O_21_ ceramics maintain the assumed chemical composition. The technology used allowed for the production of materials that are homogeneous in terms of chemical composition and which maintain their usual stoichiometry.

### 3.3. Impedance Spectroscopy

Impedance spectroscopy (IS) is a technique that analyzes the AC response of a system to a sinusoidal perturbation. The results allow us to calculate the impedance as a function of the perturbation frequency and represent it in terms of complex impedance (Z*). The impedance studies presented here were conducted at a temperature range of T = (475–800) K (with a ∆T = 10 K step) and a frequency range of f = 20 Hz–2 MHz. 

**Figure 3:**
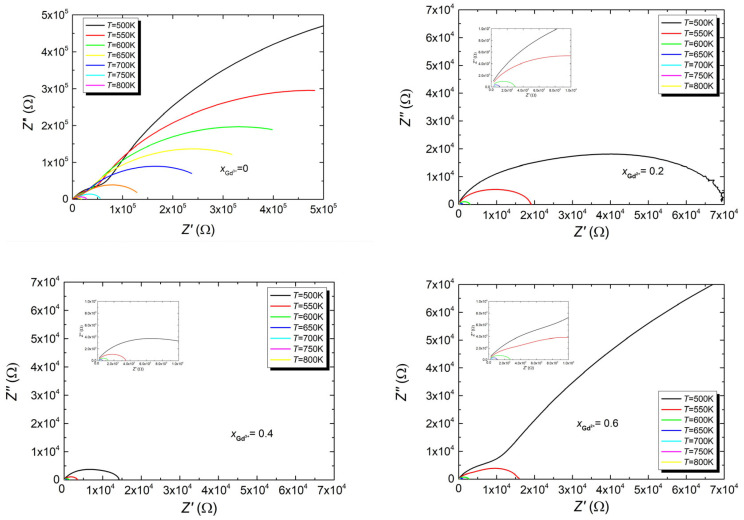
The characteristics of the dependence of the imaginary part of impedance (Z″) on the real part of impedance (Z’) of Bi_7-x_Gd_x_Fe_3_Ti_3_O_21_ ceramic materials for x = (0, 0.2, 0.4, 0.6).

It is commonly known that dielectric spectroscopy is extremely susceptible to random disturbances that may not be immediately evident in the impedance spectrum. Verifying the consistency of recorded measurements is crucial to ensure that reliable results are obtained from the impedance data analysis. For this purpose, the Kramers–Kronig (K–K) equations were used to test measurement data quality [36]. Assessment of the results showed high measurement compliance with the K–K calculations. The value of the “chi-square” parameter was obtained within the range χ^2^ = 2 × 10^−5^–6 × 10^−7^. Thus, the good quality of the measurements was confirmed, and the next analysis steps were deemed legitimate. In the next analysis stage, Nyquist plots were plotted for all discussed materials (Figure 3).

In an ideal ceramic material, the Nyquist plot typically consists of two semicircles [37]. The smaller semicircle can be attributed to the grain impedance response, while the larger one results from the presence of the free space charge accumulating at the grain boundaries and inducing polarization [38,39]. The shape of the arcs associated with the grains and the grain boundaries indicates the type of relaxation mechanism occurring in the grains and the grain boundaries. Thus, each ideal semicircular arc with its center on the Z′ axis indicates the presence of a single relaxation process described by a single relaxation time. Additionally, in the case of grains, the ideal semicircle associated with them indicates their homogeneity [39]. In the Nyquist plots observed in the case of base Bi_7_Fe_3_Ti_3_O_21_ ceramics, both arcs are not perfect semicircles, indicating the occurrence of several relaxation processes and the distribution of relaxation times associated with this fact. Furthermore, the semicircles are flattened, which is associated with the occurrence of significant grain inhomogeneity, confirmed by the SEM studies (smaller arcs) and the charge diffusion processes occurring at the grain boundaries (larger arc). The Nyquist plots drawn for ceramics containing gadolinium impurities in quantities of x = 0.2 and x = 0.4 are characterized by a single, heavily distorted semicircle, while in the case of the highest modifier concentration at low temperatures, two semicircles appear again. Selecting an electrical equivalent circuit was essential for further analysis of the obtained results. In the case of unmodified ceramics, the shape of the Nyquist plot significantly facilitated this task.

**Figure 4:**
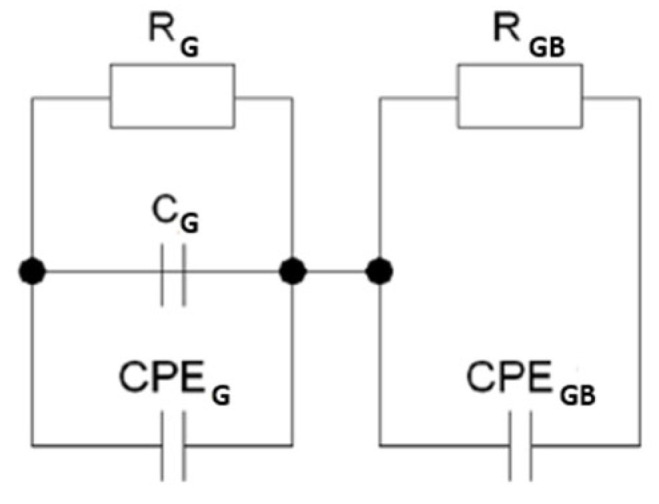
The equivalent circuit used for describing the unmodified Bi_7_Fe_3_Ti_3_O_21_ ceramics.

It is well known that this type of relationship describes an electrical equivalent circuit consisting of a double Voigt element (RC) corresponding to two regions, namely the grain interior and the grain boundaries [40]. However, since the graphs presented in Figure 3 do not represent full semicircles but only arcs of circles with reduced centers (relative to the abscissa axes), the capacitance related to the electrical properties of the grain boundaries was replaced by the capacitance of the CPE element with a constant phase. In addition, a second CPE element was added to the branch connected to the grains (Figure 4). However, the equivalent circuit designed in this way did not provide good fitting results. Therefore, in accordance with previous reports [41,42,43], we introduced an additional capacitance to the branch describing the grain behavior. This modification significantly improved the fitting parameters.

**Figure 5:**
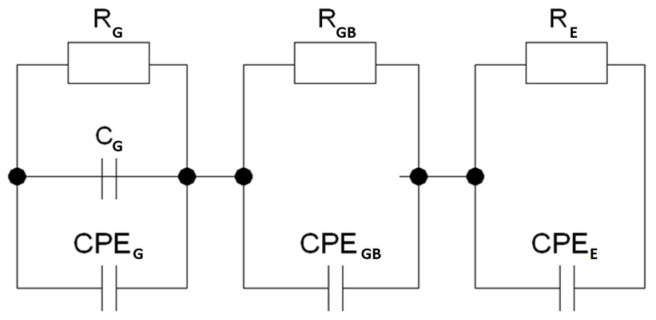
The equivalent circuit used for describing the gadolinium-ion-modified Bi_7-x_Gd_x_Fe_3_Ti_3_O_21_ for x = (0.2, 0.4, 0.6) ceramics.

In the case of gadolinium-ion-modified materials, the substitute electrical system required significant modification. Specifically, achieving good-quality matching necessitated the addition of an additional RCPE element to the aforementioned system (Figure 5). This element describes the near-electrode region, which evidently plays a more significant role in samples doped with gadolinium ions than it did in the case of the base ceramic (Figure 5). 

**Figure 6:**
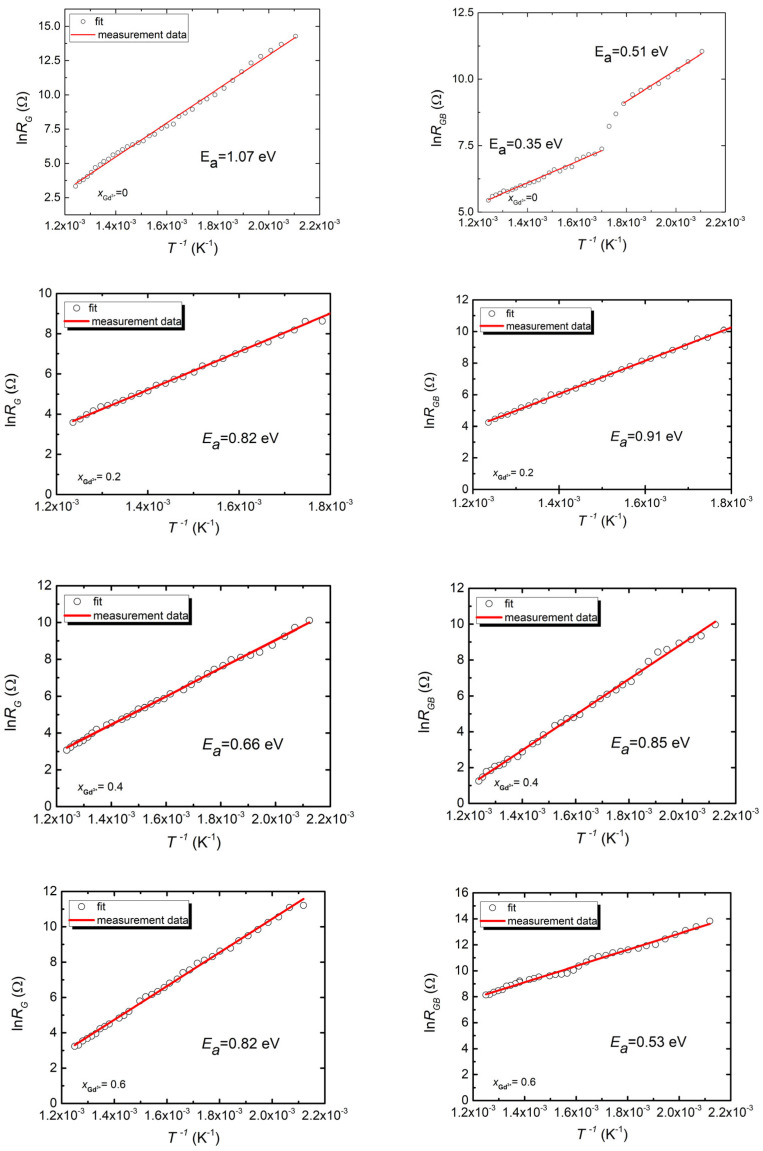
The dependence of the lnR_G_ and lnR_GB_ obtained from the analysis of impedance spectra as a function of reciprocal temperature in Bi_7-x_Gd_x_Fe_3_Ti_3_O_21_ x = (0, 0.2, 0.4, 0.6) ceramics.

The proposed modification of the substitute system allowed for the achievement of good-quality matching, resulting in obtaining resistance and capacitance values for the grains and the grain boundaries. The obtained fitting results show that the grain resistance is lower in all the materials discussed than the resistance at the grain boundary. This difference is especially noticeable at higher temperatures, and gradually decreases as the temperature decreases. This behavior is not uncommon in ceramic materials. A similar relationship is observed in materials such as SrTiO_3_ [44,45] and BaTiO_3_ [46]. According to the study authors, this is related to the presence of spatial charge layers at the grain boundaries. In these layers, the distribution of charge carriers (electrons or ions) changes due to the discontinuity at the boundary, which leads to the formation of potential barriers that impede the movement of charge carriers. This behavior results in an observed increase in the resistance of the grain boundaries compared to the grain interiors. The fitting values of the grain and the grain boundary resistance for all investigated temperatures are presented in the form of natural logarithm dependencies versus reciprocal temperature in Figure 6.

The dependencies describing the temperature changes in grain resistance have a linear appearance for all considered materials, indicating that an activated form of the conduction processes is occurring in these microstructural elements. The application of the commonly known Arrhenius equation allowed for the determination of the activation energy of these processes. In the case of the undoped material, this energy is 1.07 eV. The value of activation energy obtained for the gadolinium-modified materials shows a tendency to decrease. For a gadolinium concentration of x_Gd_ = 0.60, energy activation is equal to 82 eV. The relationship lnR_GB_(1/T) for the undoped ceramic has a more complex character; namely, it can be represented by two straight lines with different slopes, indicating the presence of two conduction processes characterized by different activation energies. The introduction of doping organizes the grain boundary conduction process. There is only one conduction process in the doped ceramic with its characteristic activation energy, which decreases with increasing dopant concentration.

In the final step of analyzing the obtained results, the total AC conductivity was determined based on Equation (2):

(2) $$\sigma_{AC}=\frac{d}{S}\cdot \frac{Z^{\prime }}{{Z^{\prime }}^{2}+{Z^{\prime \prime }}^{2}}\;$$

where:

*S*—electrode surface;
*d*—sample thickness.

In the presented dependencies, two regions are visible. The first one covers the low-frequency range. This is the region where DC conductivity, associated with the movement of charge carriers, dominates, and the dispersion dependency is almost zero. This trend changes, starting from a certain frequency known in the literature as the hopping frequency. Above this frequency, AC conductivity in the material comes into play, which is responsible for the so-called hopping conduction mechanism. This mechanism involves the hopping of charges between the minima of the spatially varying potential of the crystal lattice. This mechanism leads to an increase in conductivity and causes strong relaxation effects [47]. Based on the research presented in [48], this effect can be attributed to the hopping of polarons between the iron ions in a lower valence state (Fe^2+^) and the iron ions in a higher oxidation state (Fe^3+^).

**Figure 7:**
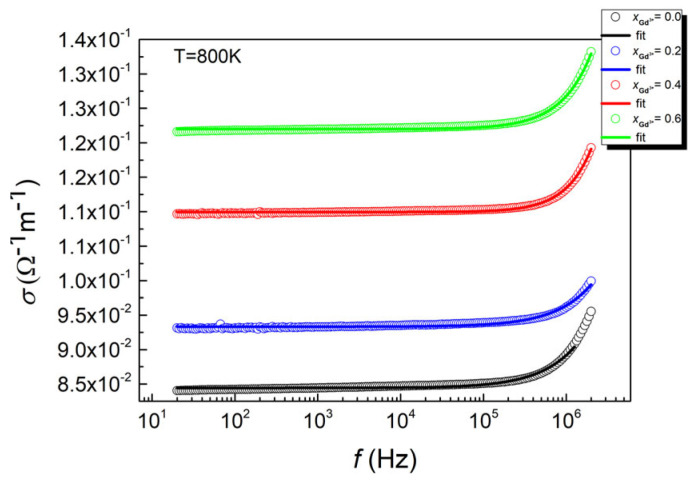
The variation in the total AC conductivity (σ_AC_) as a function of frequency at T = 800 K for the undoped material and that with gadolinium.

The shape of the σ_AC_(f) dependencies suggests the possibility of describing them with Jonscher’s exponential law [49] (3):

σ(f) = σ_DC_ + Aω^n^σ(f) (3)

where σ_DC_ describes the DC contribution, and the product *Aω*^*n*^ relates to AC conductivity, with A being a parameter dependent on temperature and associated with the polarizability of the material. The exponent *n* represents the interaction between the current carriers and the crystal lattice and takes values ranging from 0 to 1. Attempts to fit Jonscher’s law to the experimental data presented in Figure 7 were successful. The temperature dependence of the conductivity was determined.

**Figure 8:**
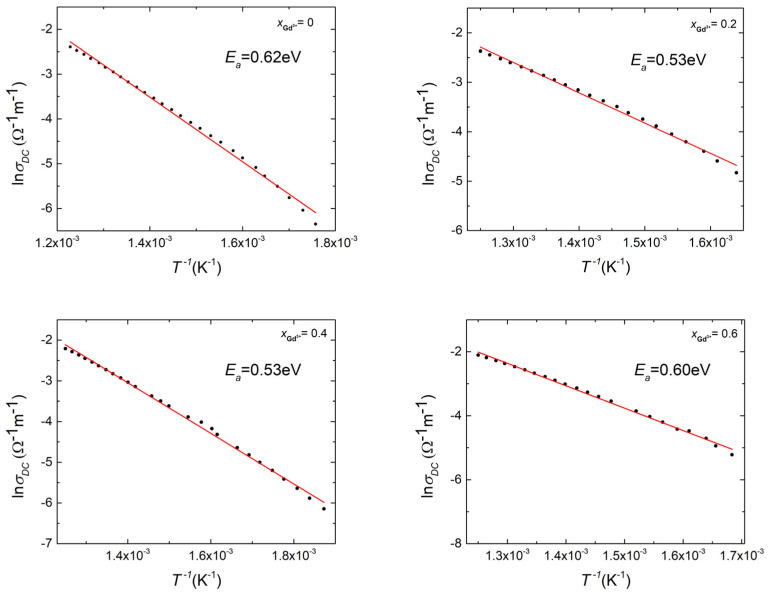
The dependence of DC conductivity, determined based on Jonscher’s law, on the inverse temperature of Bi_7-x_Gd_x_Fe_3_Ti_3_O_21_ x = (0, 0.2, 0.4, 0.6) ceramics.

The value of σ_DC_ increases with rising temperature, and the linear nature of the presented dependencies indicates the activating nature of the conduction processes occurring in the discussed ceramic materials (Figure 8). Based on the Arrhenius relationship [50], the activation energy of this process was determined to be *E*_*a*_ = 0.62 eV for the undoped material, decreasing to 0.53 eV with a gadolinium content equal to x = 0.2 and x = 0.4. This value indicates the significant influence of doubly ionized oxygen vacancies formed during the technological process on DC conductivity. Namely, during the sintering process, the following reaction occurs in the material (4):

(4) $$O_{O}^{x}=\frac{1}{2}O_{2}+2{Fe}_{Fe}^{\prime }+V_{\ddot{O}}$$

where *Fe*_*Fe*_′ represents the *Fe*^2+^ ion [51]. At sufficiently high temperatures, the mentioned oxygen vacancies are responsible for the creation of conducting electrons through ionization processes which could be described by Equations (5) and (6):

(5) $$V_{0}=V_{0}^{\prime }+e^{\prime }$$

(6) $$V_{0}^{\prime }=V_{0}^{\prime \prime }+e^{\prime }$$

The electrons become conducting electrons due to thermal activation energy. The activation energy of single ionized oxygen vacancies falls in the range of 0.3–0.6 eV, whereas for doubly ionized ones, the activation energy is equal to 0.7–1.2 eV [52]. According to A. Pelaiz-Barranco et al. [53], electrons created in this way may be captured by Fe^3+^ in consonance with Equation (7),

(7) $${Fe}^{3+}+e\Leftrightarrow {Fe}^{2+}$$

and start hopping between neighboring iron ions (Fe^2+^) and iron ions in a higher oxidation state (Fe^3+^) [51,53]. Such hopping activity is the essence of hopping conductivity.

## 4. Conclusions

The research material used was produced via a solid-state synthesis reaction using free sintering in an air atmosphere. Sublattice A (in place of bismuth ions) was modified with homovalent gadolinium ions for mole fractions of x = (0, 0.2, 0.4, 0.6). The obtained ceramic materials were subjected to XRD measurements, with the results confirming the single-phase nature of Bi_7-x_Gd_x_Fe_3_Ti_3_O_21_ ceramics. The SEM investigations show that the microstructure of undoped ceramics has a platelet–like structure, which is characteristic of the family member material Bi_m+1_Fe_m–3_Ti_3_O_3m+3_. The admixture of gadolinium changed the microstructure observed in the basic material. Namely, the dominance of large, plate-like grains disappeared in favor of smaller ones with rounded edges. The obtained impedance spectra indicate the existence in the undoped ceramics of a double relaxation phenomenon, which is attributed to the grains and grain boundaries. The admixture of gadolinium additionally activated the near-electrode areas, whose contributions were taken into account during equivalent circuit selection and the fitting process. The calculated frequency dependencies of AC conductivity obey Jonscher’s law. The detailed analysis of the mentioned dependencies points to the significant influence of doubly ionized oxygen vacancies formed during the technological process on DC conductivity.

## Figures and Tables

**Table 1:** The complete set of final parameters of the Rietveld refinement for Bi_7-x_Gd_x_Fe_3_Ti_3_O_21_ powders: *χ*^2^—chi-squared parameter, R_b_—Bragg R-factor, RF—unweighted R-factor, R_p_—least squares residuals factor, R_wp_—weighted residue factor, R_exp_—expected R-factor, and GoF—goodness of fit.

Bi_7-x_Gd_x_Fe_3_Ti_3_O_21_	*χ*^2^ [33]	R_b_	RF	R_p_	R_wp_	R_exp_	GoF
x							
0.0	2.34	1.14	1.48	4.86	6.34	4.14	1.530
0.2	1.99	4.28	2.49	6.63	8.55	6.09	1.411
0.4	1.68	1.36	0.976	5.17	6.64	5.13	1.296
0.6	1.47	1.30	2.26	5.45	6.95	5.73	1.212

## Data Availability

Data are contained within the article.
